# Does an Immigrant Health Advantage Exist Among US Whites? Evidence from a Nationally-Representative Examination of Mental and Physical Well-Being

**DOI:** 10.1007/s10903-024-01607-4

**Published:** 2024-06-03

**Authors:** Jen’nan G. Read

**Affiliations:** 1 Department of Sociology, Duke University, 417 Chapel Drive, Durham, NC 27708, USA; 2 Global Health Institute, Duke University, Durham, NC 27708, USA

**Keywords:** Immigrant health, White immigrants, non-Hispanic Whites, Mental health, Physical health

## Abstract

This study examines whether an immigrant health advantage exists among US Whites, a group often used as a reference category in research on racial and ethnic health disparities. Using recent data from the National Health Interview Survey (2019–2022), I disaggregate non-Hispanic White adults (*n* = 41,752) by nativity status and use logistic regression models to assess differences in six measures of mental and physical health. The analysis includes self-reported conditions (depression, anxiety, fair/poor self-rated health) and diagnosed conditions that require interaction with the healthcare system (hypertension, diabetes, and chronic obstructive pulmonary disease, COPD). Foreign-born Whites have a significantly lower prevalence of each health outcome relative to US-born Whites. The immigrant health advantage remains significant for depression, anxiety, fair/poor health (i.e., self-reported conditions) and diagnosed hypertension, after adjusting for sociodemographic and healthcare characteristics. In contrast, the inclusion of these explanatory factors reduces the nativity gap in diagnosed diabetes and COPD to non-significance. Overall, the results indicate important variation in health among Whites that is missed in studies that focus on US-born Whites, alone. Scholars must continue to monitor the health of White immigrants, who are projected to grow to 20% of the US immigrant population in the years to come.

## Introduction

The health and well-being of immigrants is a topic of growing public health concern. In 2022, immigrants made up 13.9% (46.2 million) of the US population, up from 7.9% in 1990 and more than double in absolute size (19.8 million) [1, 2]. Immigrants are also diversifying the composition of US racial and ethnic populations, which has implications for population health and the healthcare system [3, 4]. To date, the majority of evidence on immigrant health has derived from research on the largest immigrant group, Hispanics (42%), and to a lesser extent, on Asian (27%) and Black (11%) immigrants [5–9]. The general finding in the literature is one of better health among immigrants relative to their US-born counterparts, which is often referred to as the “healthy immigrant effect” or “immigrant health advantage” [10–13].

**Fig. 1:**
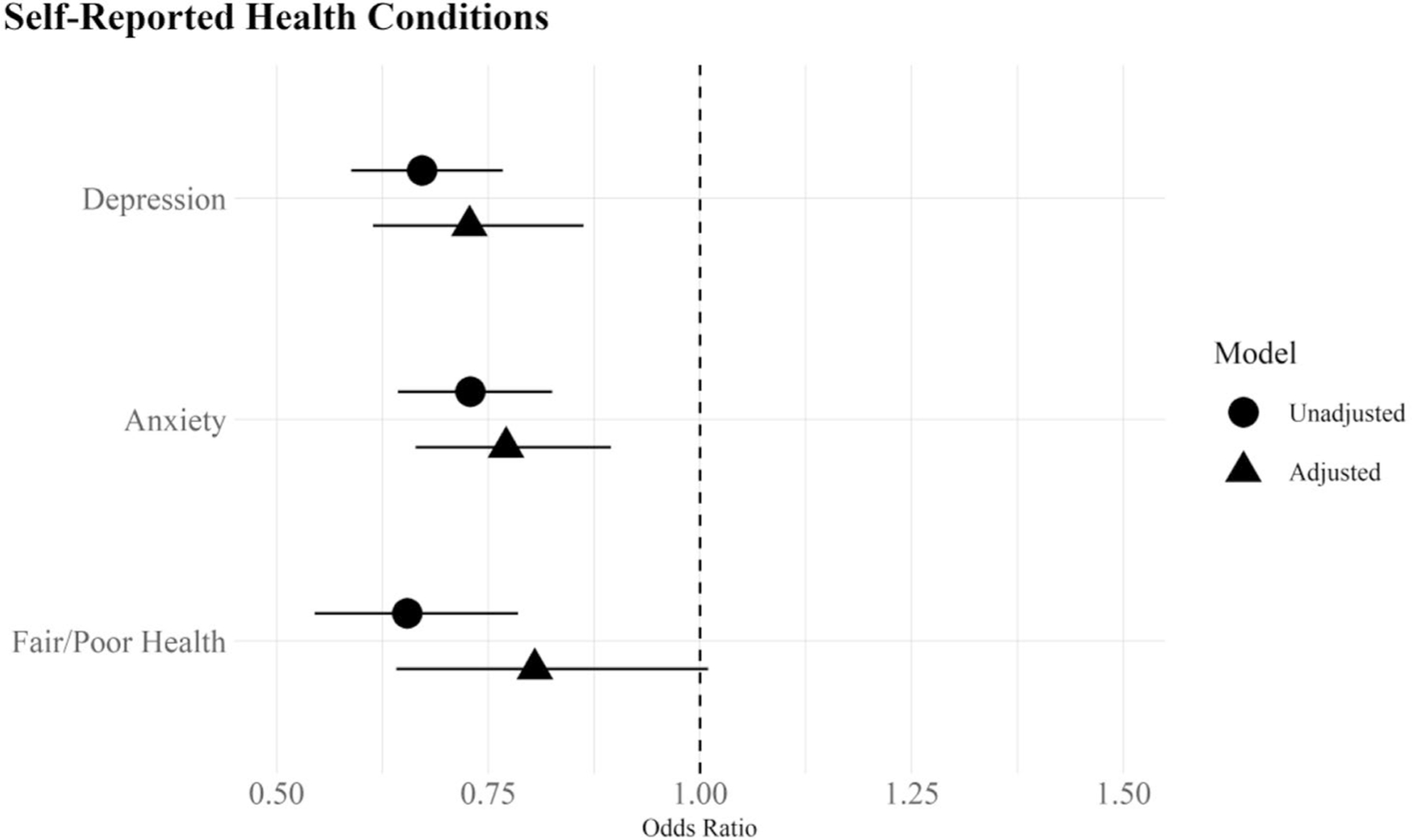
Odds ratios of self-reported health conditions by nativity status with 95% confidence intervals *Note*: Odds ratios derived from logistic regression models in Table 2, Panel A. The unadjusted models include nativity status (Model 1) and the adjusted models include all covariates (Model 2)

Compared to research on racial and ethnic minorities, surprisingly little is known about the health of non-Hispanic White immigrants (hereafter White). This is an important oversight for several reasons. First, foreign-born Whites make up 17% (7.6 million) of the US immigrant population and are projected to grow to 20% (15.6 million) by 2060 [14, 15]. Not only will their numbers contribute to patterns in immigrant health, but also to patterns in overall population health via growth in the second and third generations [15]. Second, Whites are ethnically diverse and defined by the US Census as persons who trace their ancestries to “any of the original peoples of Europe, the Middle East, and North Africa” [16, 17]. When the federal government established standards for racial and ethnic categories^1^ in 1977, western European Whites comprised over 70% of the total US population and 60% of the foreign-born White population [18–20]. Today, those numbers have dropped to roughly one-third each, while Middle Eastern and eastern European immigrants—who are also classified as White—have grown to comprise over one-half (53%) of the foreign-born White population (author’s calculations from US Census data, see Fig. 1 online supplement).

The majority of immigrants from the Middle East and eastern Europe have arrived in the US since 1990, after the Iran-Iraq War (1980–89), fall of the Former Soviet Union (1991), and beginning of the first Gulf War (1991) [20]. They are linguistically, phenotypically, and culturally more diverse than their western European predecessors, and many have migrated under challenging circumstances due to political and civil unrest in their countries of origin [21–23]. Their contexts of migration have led to lower levels of health selection relative to immigrants arriving from more economically and politically stable regions [23–25]. Once in the US, these newer immigrants are incorporated into a racial stratification system that classifies them as “White,” even if they do not identify or pass as White [26, 27]. As a result, health disparities within these populations may be overlooked when aggregated into the broad White category.

Studies that disaggregate White ethnic subgroups, such as Arab Americans, have indeed found higher rates of diabetes [28, 29], heart disease [30], disability [31], cognitive limitations [32], psychological distress [11], and poorer self-rated health [33] than US-born Whites, even after accounting for compositional differences in socio-demographic characteristics (i.e., factors that might account for health disparities). Immigrants from the Former Soviet Union, who are also classified as White, likewise report higher rates of hypertension [34], disability [21, 35], and poorer self-rated health [10, 23] when compared to US-born Whites. Together, these studies suggest that the healthy immigrant effect found for other racial/ethnic groups may be weaker among Whites, at least for some White subgroups.

While informative, research on specific ethnic subgroups paints a partial picture of White immigrant health. Studies that compare all foreign-born Whites to US-born Whites are limited, and even fewer have examined multiple mental and physical health outcomes in the same analytic framework. Thus, the extent to which US- and foreign-born Whites differ across a range of outcomes is unknown. Less is also known about compositional variation among White immigrants on factors related to health, such as poverty and access to healthcare. Evaluating the compositional characteristics of Whites could provide a more complete understanding of the mechanisms driving observed health disparities. The current study addresses these questions and contributes to a nascent literature on White immigrant health. Using the most recently available data from the National Health and Interview Survey (2019–2022), I disaggregate Whites by nativity status and assess differences in six measures of mental and physical health. I center the analysis on three interrelated research questions:

RQ1) To what extent is there a nativity gap in health among Whites (i.e., do the foreign-born. experience a health advantage over the US-born)?;

RQ2) Does the nativity gap vary across measures of mental and physical health?;

RQ3) Do compositional differences in sociodemographic and healthcare factors.

account for observed disparities in health between US- and foreign-born Whites?

## Methods

### Data and Measures

This study uses merged data from the 2019 to 2022 National Health Interview Survey (NHIS).^2^ The data were obtained through the University of Minnesota’s Integrated Public Use Microdata Series (IPUMS) and include pooled samples from the 2019 to 2022 files with integrated sampling weights. The analytic sample includes US-born (*n* = 39,468) and foreign-born (*n* = 2,284) non-Hispanic White adults ages 25 to 64.^3^ The data are publicly available and contain no identifying information on respondents, thus deemed exempt from Institutional Review Board (IRB) approval.

The dependent variables include three *self-reported health conditions* (where respondents evaluated their own health status) and three *diagnosed health conditions* (where respondents had to interact with the healthcare system to receive a diagnosis). The *self-reported conditions* are depression, anxiety, and self-rated health. *Depression* and *anxiety* were coded to capture respondents who reported feeling depressed or anxious/worried/nervous on a daily, weekly, or monthly basis (reference = never or a few times a year), and *self-rated health* identified those who reported fair or poor health (reference = excellent, very good, or good). The *diagnosed health conditions* are *diabetes*, *hypertension* (or high blood pressure), and *chronic obstructive pulmonary disease* (COPD). Each outcome is associated with several leading causes of death (e.g., heart attack, stroke) [36] and requires access to healthcare to receive a diagnoses [37, 38]. Responses were coded to identify respondents who had been diagnosed with each condition (reference = not diagnosed).

The key independent variable is *nativity* (reference = US-born). I also include several sets of covariates that might account for observed heterogeneity in health [10]. *Socioeconomic characteristics* are captured with measures for educational attainment (reference = less than high school); employment status (reference = not currently working/not in the labor force); and poverty status (reference = 5 times or more above the poverty threshold). *Healthcare access* includes health insurance coverage (reference = no coverage); having a usual place of care (reference = no); and having seen a doctor or other healthcare professional in the past 12 months (reference = no). *Demographic* and *health behavior characteristics* are age and age squared (continuous in years); gender; marital status; presence of children in the home; US citizenship status; region of US residence; body mass index (BMI); and smoking status. I conducted several sensitivity analyses to ensure that measurement decisions did not affect the primary results (available on request).

### Analytic Strategy

**Fig. 2:**
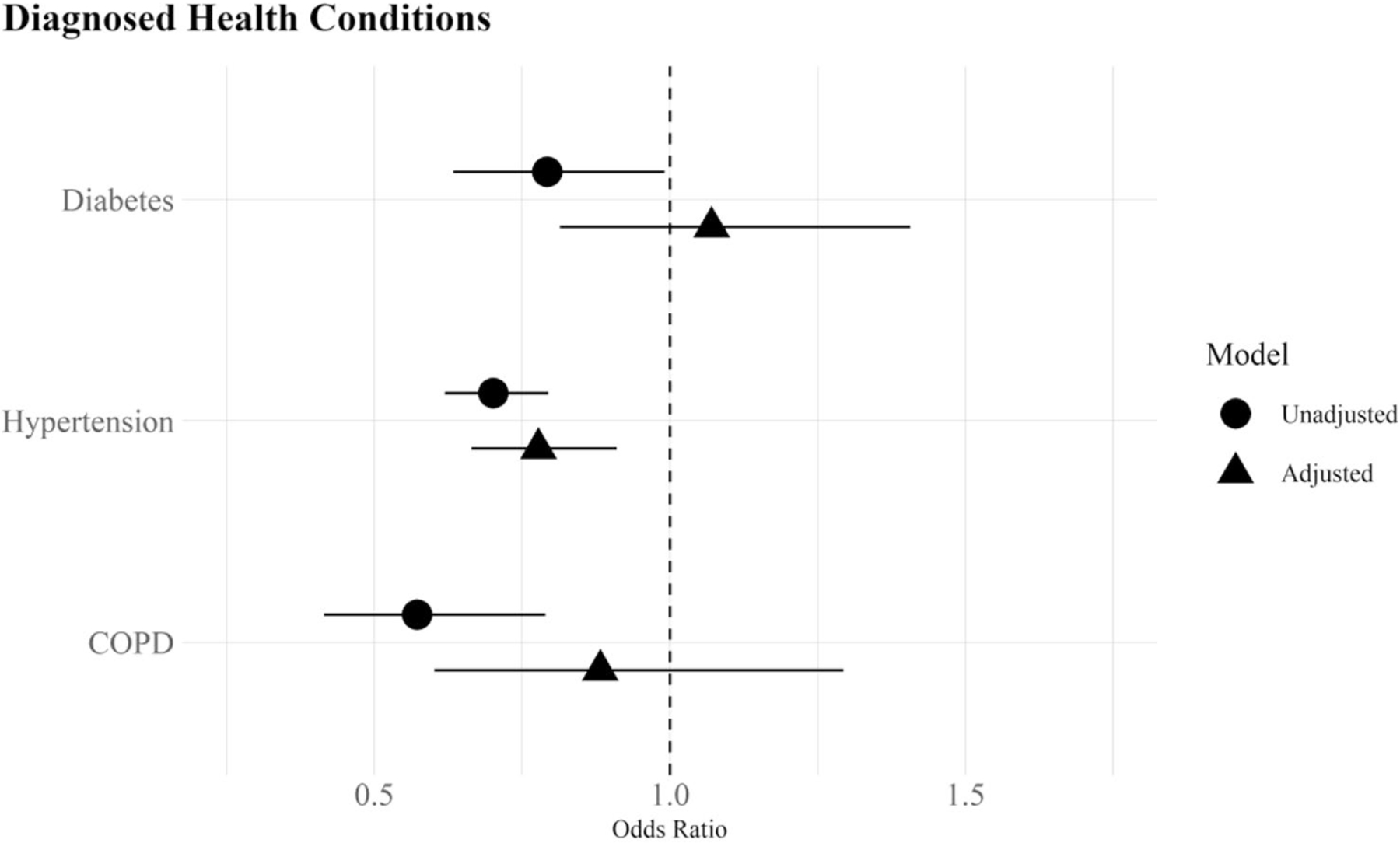
Odds ratios of diagnosed health conditions among whites by nativity status with 95% confidence intervals, National Health Interview Survey 2019–2022 *Note*: Odds ratios derived from logistic regression models in Table 2, Panel B. The unadjusted models include nativity status (Model 1) and the adjusted models include all covariates (Model 2)

Table 1 provides descriptive statistics on the key variables of interest (Table S1, online supplement, contains all variables in the analyses). I use Pearson chi-square (χ2) and Wald tests with Rao and Scott’s second-order correction to identify significant differences between US- and foreign-born Whites across health conditions (RQ1, RQ2) and sociodemographic and healthcare factors (RQ3). I then use a series of logistic regression models to estimate health outcomes, with and without adjustment for covariates, using the Bayesian Information Criteria (BIC) to identify the best model fit. Table 2 presents the odds ratios predicting self-reported health conditions in Panel A and diagnosed health conditions in Panel B (Tables S2 and S3 include the full set of covariate estimates). Figures 1 and 2 plot the odds ratios from Panels A and B to ease interpretation of findings. All analyses were conducted using R version 4.2.2 and the survey package [39] to incorporate NHIS sample weights.

## Results

As seen in Table 1, foreign-born respondents report fewer self-reported and diagnosed health conditions than do US-born Whites, which provides initial evidence of a nativity gap in health among Whites (RQ1). Further, the prevalence of disease and size of the nativity gap varies across the six outcomes (RQ2). For self-reported conditions, one in five (19.8%) US-born Whites report experiencing depression and one in four (25%) report experiencing anxiety, compared to 14.2% and 19.5% of the foreign-born, respectively. One in ten (11.3%) US-born Whites also rate their health as “fair or poor” compared to 7.7% of foreign-born Whites. The prevalence for diagnosed conditions is highest for hypertension, with one-fourth of US-born and one-fifth of foreign-born Whites reporting being diagnosed with high blood pressure. The prevalence of diabetes and chronic obstructive pulmonary disease (COPD) are lower, as are the nativity gaps: 6.3% of US-born and 5.1% of foreign-born Whites report being diagnosed with diabetes and 4.2% and 2.5% with COPD. Though smaller, the nativity gaps for these conditions are significant.

Turning to explanatory factors, foreign-born Whites have relatively high levels of educational attainment and are bifurcated in terms of poverty status, with higher concentrations in the lowest and highest levels of poverty. There is no significant variation by nativity status for health insurance coverage, having a usual place of care, or having seen a provider in the past year. US-born Whites are more likely to be overweight or obese and be current smokers, while foreign-born Whites are more likely to be currently married, have a minor in the home, and live in the Northeast and West regions. The age and gender composition of US- and foreign-born Whites are similar, with a mean age of approximately 45 years and an equal distribution of males and females.

Table 2 presents odds ratios from logistic regression models assessing differences in self-reported (Panel A) and diagnosed conditions (Panel B). Panel A shows that the immigrant health advantage seen in Table 1 remains significant in the multivariable context and is largely impervious to adjustment for sociodemographic and healthcare factors. For example, foreign-born Whites have 37% lower odds of depression in the baseline model (OR = 0.67; CI = 0.59, 0.77) that decreases to 27% but remains significantly lower than US-born Whites in the fully adjusted model (OR = 0.73; CI = 0.61, 0.86). Changes in the nativity gap for anxiety are even smaller, decreasing from 27% lower odds in Model 1 to 23% in Model 2 (OR = 0.73; CI = 0.64, 0.83 and OR = 0.77, CI = 0.66, 0.90).

Changes in the odds of fair/poor self-rated health are most affected by the inclusion of covariates; in the unadjusted Model 1, foreign-born Whites have a 35% lower odds of reporting fair/poor health (OR = 0.65, CI = 0.55, 0.79) that drops in size and significance after adjustment for covariates (OR = 0.81, CI = 0.64–1.01). Figure 1 eases interpretation by graphing the unadjusted and adjusted odds ratios for each condition. As seen in the figure, foreign-born Whites have lower unadjusted odds of depression, anxiety, and fair/poor self-rated health that remain significantly lower than US-born Whites after the inclusion of control variables. The biggest change is for self-rated health, where the inclusion of covariates reduces the foreign-born advantage by nearly one-half. The overall pattern in Fig. 1 indicates that compositional heterogeneity in sociodemographic and healthcare factors, alone, do not fully account for the nativity gap in self-reported outcomes (RQ3).

For diagnosed medical conditions (Panel B), the immigrant advantage for diabetes and COPD seen in Table 1 disappears and is no longer statistically different from that of US-born Whites in the fully adjusted model (OR = 1.07; CI = 0.63, 1.41 for diabetes; OR = 0.88; CI = 0.60, 1.29 for lung disease). Hypertension is the only diagnosed condition where foreign-born Whites have a lower odds of diagnoses (OR = 0.70; CI = 0.62, 0.79) that remains robust after adjustment for covariates (OR = 0.78; CI = 0.66, 0.91). Figure 2 simplifies interpretation of the findings by graphing the results for each of the diagnosed conditions. As seen in the figure, the foreign-born advantage evidenced for self-reported health conditions (Fig. 1) is considerably smaller and not statistically different from US-born Whites, especially in the case of diabetes and COPD. Hypertension stands out in that the odds of reporting a diagnosis remains significantly lower for foreign-born Whites in the fully adjusted analysis.

## Discussion

Inequality research often uses US-born, non-Hispanic Whites as the reference group to measure US health disparities, with less attention paid to diversity *among* Whites, particularly White immigrants. White immigrants make up a sizable portion (17%) of the US foreign-born population, yet are surprisingly absent in the overwhelming majority of research on US racial/ethnic inequality [4], immigrant health [24], and even in studies that examine Whiteness and White racial identity [40]. The current study addresses these oversights by disaggregating US Whites by nativity status and examining differences in six measures of health that tap into dimensions of mental and physical well-being. I used one of the only nationally-representative datasets available (NHIS) that contains detailed information on nativity and health status, with large enough sample sizes of White immigrants for analyses.

The results found an overall pattern of better health among White immigrants compared to US-born Whites, which is similar to findings of an ‘immigrant health advantage’ for other racial/ethnic groups but counter to studies that find little to no health advantage for specific White subgroups, such as Arab Americans [33]. One plausible explanation is that the foreign-born White category contains other large ethnic groups, namely Europeans, which contributes to overall averages in White immigrant health. These averages, in turn, can mask within-group heterogeneity among White immigrants. Indeed, prior studies that disaggregate White immigrants by region of birth lend support to this possibility [20, 21]. Unfortunately, the NHIS dropped the region of birth question in 2018, which I discuss in more detail below.

Beyond the general pattern of better health, there was diversity in the size of the health gap between US- and foreign-born Whites depending on the health measure in question. The efficacy of sociodemographic and healthcare factors in explaining health gaps also varied by health condition. For self-reported conditions–depression, anxiety, and fair/poor self-rated health–foreign-born Whites had a health advantage over US-born Whites that remained significant after adjustment for covariates. In fact, the odds of reporting better health were remarkably similar in the baseline and fully adjusted models, suggesting that compositional differences in sociodemographic and healthcare characteristics were not responsible for health gaps between foreign- and US-born Whites, at least not for these health conditions.

Findings for diagnosed health conditions, which required interaction with the healthcare system, were slightly more complex. First, the prevalence of diabetes and chronic obstructive pulmonary disease (COPD) were relatively low when compared with hypertension, likely due to the age composition of the sample that excluded later stages of the life course when disease prevalence is highest. Second, the inclusion of covariates reduced the nativity gap to non-significance for diabetes and COPD but did little to alter the gap in diagnosed hypertension. Symptoms associated with diabetes and COPD are more noticeable than those for hypertension, and thus may motivate health-seeking behaviors for treatment that reduce between-group disparities. Findings for hypertension were similar to those for depression, anxiety, and fair/poor self-rated health (i.e., self-reported health conditions) in that standard explanations for health disparities did not account for the better health of foreign-born Whites. These results suggest that other mechanisms not observed in the current study may be contributing to health disparities in conditions that are harder to detect (e.g., hypertension, depression).

This study is not without limitations. First, I was unable to disaggregate White immigrants by region or country of birth due to the removal of region of birth in the 2019 NHIS survey redesign. I attempted to account for this shortcoming by using duration of US residence and immigrant arrival cohort as proxy measures for region of birth but found that sample sizes were too small for meaningful analyses (e.g., 71% of foreign-born whites had resided in the US for 15 years or more, analyses available on request). It is worth noting that the redesign also affects studies that used restricted data to examine specific country-of-origin groups. Second, the current study focused on non-Hispanic Whites and excluded a smaller but growing number of Whites who identify as Hispanic. The decision was purposeful and aimed at disrupting the long-standing practice of using non-Hispanic Whites as a benchmark for measuring US racial/ethnic health disparities [41]. Moving forward, research will need to consider the role of Hispanic ethnicity in analyses of White health, especially as overall growth in the US White population is projected to result, in part, from increases in Whites of Hispanic ethnicity [15].

## Conclusions

White immigrants are projected to grow to 20% of the US foreign-born population and 7% of the White population by 2060 [15], thus understanding diversity among Whites is crucial for knowledge on racial, ethnic, and immigrant health disparities. This study focuses on this question and makes several contributions to existing research. By focusing on non-Hispanic Whites, I extend recent work that underscores ethnic heterogeneity within US racial groups, most of which has concentrated on racial and ethnic minorities [4]. I also challenge the practice of using non-Hispanic Whites as a reference group to measure racial gaps in health. The practice emerged in a time of greater ethnic homogeneity among Whites (i.e., western European) and should be used with caution moving forward, especially when examining trends over time. Studies that fail to consider compositional changes among Whites could yield biased or misleading estimates of progress (or the lack thereof) toward racial/ethnic equity. Policy decisions that are based on racial comparisons are likewise implicated in findings from this study. Future research will need to continue monitoring the composition of US Whites to better specify the conditions that contribute to within- and between-group racial health disparities.

## Supplementary Material

Supplemental figures and tables

## Figures and Tables

**Table 1:** Descriptive statistics for us whites by nativity status, ages 25–64, National Health Interview Survey, United States, 2019–2022

	US-Born (*N* = 39,468)	Foreign-Born (*N* = 2,284)	*p*-value
Self-reported health conditions			
Depression (monthly or more)	19.8	14.2	< 0.001
Anxiety (monthly or more)	25.0	19.5	< 0.001
Fair/poor self-rated health	11.3	7.7	< 0.001
Diagnosed health conditions			
Diabetes	6.3	5.1	< 0.041
Hypertension	26.5	20.1	< 0.001
Chronic obstructive pulmonary Disease (COPD)	4.2	2.5	< 0.001
Educational attainment			< 0.001
Less than high school	4.7	4.8	
High school diploma	24.5	18.4	
Some college or Associate’s degree	29.9	21.9	
Bachelor’s degree or more	41.0	54.9	
Poverty level			< 0.001
Below poverty threshold	5.9	6.7	
1 to 1.99 x’s above	11.7	12.0	
2 to 2.99 x’s above	13.9	12.0	
3 to 3.99 x’s above	14.1	11.3	
4 to 4.99 x’s above	13.1	12.2	
5 x’s or more above	41.3	45.8	
Employment Status			< 0.016
Not Working	21.8	22.4	
Working less than full-time	10.1	12.0	
Working full-time	68.2	65.6	
Healthcare Coverage			< 0.056
No Coverage	8.4	10.1	
Public Health Coverage	13.0	13.0	
Private Health Coverage	75.4	74.4	
Other Coverage	3.2	2.6	
Has a Usual Place for Care	89.8	88.5	< 0.120
Has Seen a Provider in Past Year	82.2	81.4	< 0.400
BMI (body mass index)			< 0.001
Normal Weight	27.9	35.2	
Underweight	0.6	1.0	
Overweight	32.2	36.3	
Obese	31	21.9	
Missing	8.3	5.6	
Smoking Status			< 0.001
Never	58.0	61.2	
Former	25.5	25.8	
Current	16.4	13	
Sex			< 0.700
Male	49.9	50.5	
Female	50.1	49.5	
Age (mean, years)	45.2	45.6	< 0.001
US Citizen	100.0	69.6	< 0.001
Marital status			< 0.001
Married	62.1	71.4	
Divorced/separated/widowed	15.9	14.4	
Never Married	21.9	14.3	
Child < 18yrs present in home	37	41.3	< 0.002
Length at current house/apartment			< 0.001
Less than 1 year	11.5	13.2	
1 to 3 years	24.5	27.6	
4 to 10 years	28.3	28.6	
11 to 20 years	20	21	
More than 20 years	15.8	9.6	
US region of residence			< 0.001
Northeast	18.5	26.5	
North Central/Midwest	27.3	17.7	
South	34.2	28.3	
West	20	27.6	

Note: Estimates are weighted, sample sizes are unweighted

*p*-values are calculated by Wald Tests for continuous variables and Chi-Squared Tests with Rao and Scott’s Second-Order Correction for categorical variables

**Table 2:** Multivariable results for self-reported and diagnosed health conditions among whites by nativity status, ages 25–64: National Health Interview Survey, United States, 2019–2022

*Panel A: Self-Reported Conditions*						
	Depression OR (95% CI)		Anxiety OR (95% CI)		Fair/Poor Health OR (95% CI)	
	Model 1	Model 2	Model 1	Model 2	Model 1	Model 2
Nativity (US-Born)	1.00	1.00	1.00	1.00	1.00	1.00
Foreign-Born	0.67**	0.73**	0.73**	0.77**	0.65**	0.81ϯ
	(0.59–0.77)	(0.61–0.86)	(0.64–0.83)	(0.66–0.90)	(0.55–0.79)	(0.64–1.01)
*Panel B: Diagnosed Conditions*						
	Diabetes OR (95% CI)		COPD OR (95% CI)		Hypertension OR (95% CI)	
	Model 1	Model 2	Model 1	Model 2	Model 1	Model 2
Nativity (US-Born)	1.00	1.00	1.00	1.00	1.00	1.00
Foreign-Born	0.79*	1.07	0.57**	0.88	0.70**	0.78**
	(0.63–0.99)	(0.81–1.41)	(0.62–0.79)	(0.60–1.29)	(0.42–0.79)	(0.66–0.91)

Odds ratios with 95% confidence intervals in parentheses (CI) from logistic regression models. All estimates are weighted. Model 1 is the baseline model that includes nativity status, and Model 2 adjusts for education, poverty, employment, health insurance coverage, usual place for healthcare, seen a provider in past year, age, gender, US citizenship, marital status, presence of children at home, length at residence, and region of US residence.

Table S2 and Table S3 (supplements) contain coefficients for all variables in the models.

** *p* < 0.01

* *p* < 0.05

ϯ *p* < 0.10

## Footnotes

1 OMB Directive 15 defined race separately from Hispanic origin and established five standard categories for collecting and analyzing: White, not Hispanic; Black, not Hispanic; Hispanic, any race; Asian or Pacific Islander; and American Indian or Alaska Native.

2 In 2019, the NHIS underwent a major redesign with a new structure and content that prevents comparisons with prior survey years.

3 Focusing on this age group avoided potential confounders associated with retirement or enrollment in higher education, and sensitivity analyses with other age groupings yielded substantively similar findings.
