# Supplementary material for: Does an Immigrant Health Advantage Exist Among US Whites? Evidence from a Nationally-Representative Examination of Mental and Physical Well-Being
Source: J Immigr Minor Health. 2024 Jun 3;26(5):878–86. doi: 10.1007/s10903-024-01607-4 (PMC11412786; doi:10.1007/s10903-024-01607-4)
Supplement: Supplementary file 1 — Supplementary Material 1 [file 10903_2024_1607_MOESM1_ESM.docx]

**SUPPLEMENTARY FIGURE AND TABLES**


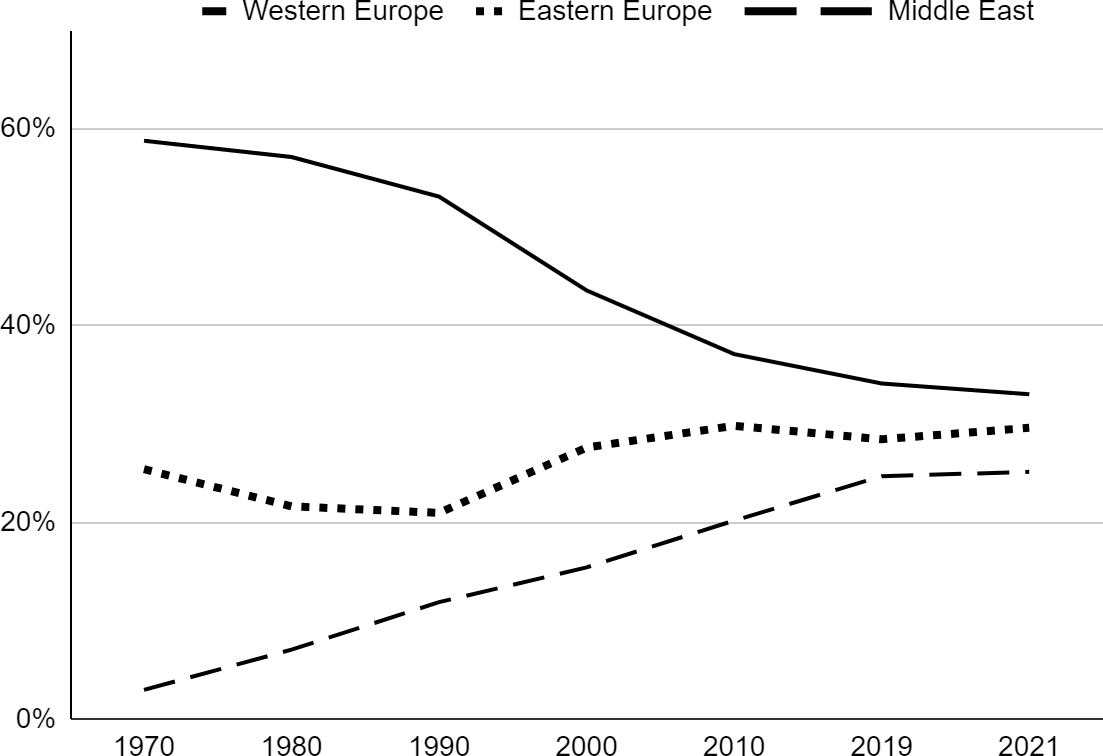


58%

33%

25%

30%

25%

3%

Figure S1. Major regions of birth for non-Hispanic White immigrants, 1970 to 2021.

*Sources:* Authors’ tabulations from U.S. Census Bureau decennial census and American Community Survey, 1960–2021.

Table S1: Descriptive Statistics for US Whites by Nativity Status, ages 25-64, National Health Interview Survey, United States, 2019-2022

|  | US-Born (N = 39,468) | Foreign-Born (N = 2,284) | *p*-value |
| --- | --- | --- | --- |
| Depression (monthly or more) | 19.3 | 14.2 | <0.001 |
| Depression categories |  |  |  |
| Never | 49.9 | 60.0 |  |
| A few times a year | 30.3 | 25.8 |  |
| Monthly | 8.6 | 6.7 |  |
| Weekly | 6.6 | 4.8 |  |
| Daily | 4.6 | 2.7 |  |
| Anxiety (monthly or more) | 25.0 | 19.5 | <0.001 |
| Anxiety categories |  |  |  |
| Never | 22.2 | 27.7 |  |
| A few times a year | 30.6 | 31.9 |  |
| Monthly | 13.4 | 14.0 |  |
| Weekly | 18.0 | 14.8 |  |
| Daily | 15.7 | 11.7 |  |
| Fair/Poor self-rated health | 11.3 | 7.7 | <0.001 |
| Self-rated health categories |  |  |  |
| Excellent | 24.2 | 31.3 |  |
| Very Good | 38.7 | 38.2 |  |
| Good | 25.8 | 21.8 |  |
| Fair | 8.6 | 5.9 |  |
| Poor | 2.7 | 1.8 |  |
| Diabetes | 6.3 | 5.1 | <0.041 |
| Hypertension | 26.5 | 20.1 | <0.001 |
| COPD | 4.2 | 2.5 | <0.001 |
| Educational Attainment |  |  | <0.001 |
| Less than high school | 4.7 | 4.8 |  |
| High school diploma | 24.5 | 18.4 |  |
| Some college or Associate’s degree | 29.9 | 21.9 |  |
| Bachelor’s degree or higher | 41.0 | 54.9 |  |
| Poverty level |  |  | <0.001 |
| Below poverty threshold | 5.9 | 6.7 |  |
| 1 to 1.99 x’s above | 11.7 | 12.0 |  |
| 2 to 2.99 x’s above | 13.9 | 12.0 |  |
| 3 to 3.99 x’s above | 14.1 | 11.3 |  |
| 4 to 4.99 x’s above | 13.1 | 12.2 |  |
| 5 x’s or more above | 41.3 | 45.8 |  |
| Employment Status |  |  | <0.016 |
| Not Working | 21.8 | 22.4 |  |
| Working Less Than Full-Time | 10.1 | 12.0 |  |
| Working Full-Time | 68.2 | 65.6 |  |
| Healthcare Coverage |  |  | <0.056 |
| No Coverage | 8.4 | 10.1 |  |
| Public Health Coverage | 13.0 | 13.0 |  |
| Private Health Coverage | 75.4 | 74.4 |  |
| Other Coverage | 3.2 | 2.6 |  |
| Having Usual Healthcare Place | 89.8 | 88.5 | <0.120 |
| Doctor Visits within the Last Year | 82.2 | 81.4 | <0.400 |
| BMI Categories |  |  | <0.001 |
| Normal Weight | 27.9 | 35.2 |  |
| Underweight | 0.6 | 1.0 |  |
| Overweight | 32.2 | 36.3 |  |
| Obese | 31 | 21.9 |  |
| BMI Info Missing | 8.3 | 5.6 |  |
| Smoking Status |  |  | <0.001 |
| Never | 58.0 | 61.2 |  |
| Former | 25.5 | 25.8 |  |
| Current | 16.4 | 13.0 |  |
| Sex |  |  | <0.700 |
| Male | 49.9 | 50.5 |  |
| Female | 50.1 | 49.5 |  |
| Age (mean, yrs) | 45.3 | 45.6 | <0.001 |
| US Citizen | 100.0 | 69.6 | <0.001 |
| Marital status |  |  | <0.001 |
| Married | 62.1 | 71.4 |  |
| Divorced/separated/widowed | 15.9 | 14.4 |  |
| Never married | 21.9 | 14.3 |  |
| Child <18yrs present in home | 37.0 | 41.3 | <0.002 |
| Length at current house/apartment |  |  | <0.001 |
| Less than 1 year | 11.5 | 13.2 |  |
| 1 to 3 years | 24.5 | 27.6 |  |
| 4 to 10 years | 28.3 | 28.6 |  |
| 11 to 20 years | 20.0 | 21.0 |  |
| More than 20 years | 15.8 | 9.6 |  |
| US region of residence |  |  | <0.001 |
| Northeast | 18.5 | 26.5 |  |
| North Central/Midwest | 27.3 | 17.7 |  |
| South | 34.2 | 28.3 |  |
| West | 20.0 | 27.6 |  |

Note: Estimates are weighted, sample sizes are unweighted.

*p*-values are calculated by Wald Tests for continuous variables and Chi-Squared Tests with Rao and Scott's Second-Order Correction for categorical variables.

Table S2: Multivariable results predicting self-reported health conditions among Whites by nativity status, ages 25-64: National Health Interview Survey, United States, 2019-2022

|  | Depression  OR (95% CI) | | Anxiety  OR (95% CI) | | Fair/Poor Health  OR (95% CI) | |
| --- | --- | --- | --- | --- | --- | --- |
|  | Model 1 | Model 2 | Model 1 | Model 2 | Model 1 | Model 2 |
| (Intercept) | 0.25** | 0.07** | 0.33** | 0.08** | 0.13** | 0.00** |
|  | (0.24, 0.26) | (0.04, 0.14) | (0.32, 0.34) | (0.04, 0.15) | (0.12, 0.13) | (0.00, 0.00) |
| Nativity (US-born) |  |  |  |  |  |  |
| Foreign-Born | 0.67** | 0.73** | 0.73** | 0.77** | 0.65** | 0.81ϯ |
|  | (0.59-0.77) | (0.61-0.86) | (0.64-0.83) | (0.66-0.90) | (0.55-0.79) | (0.64-1.01) |
| Educational Attainment (< HS) |  |  |  |  |  |  |
| HS Diploma |  | 0.91 |  | 0.97 |  | 0.73** |
|  |  | (0.78, 1.07) |  | (0.84, 1.13) |  | (0.61, 0.87) |
| Some College or Associate |  | 1.10 |  | 1.16* |  | 0.65** |
|  |  | (0.94, 1.29) |  | (1.00, 1.35) |  | (0.55, 0.79) |
| College Degree or More |  | 1.25** |  | 1.30** |  | 0.51** |
|  |  | (1.07, 1.47) |  | (1.12, 1.49) |  | (0.42, 0.62) |
| Poverty (5 x’s or more above threshold) |  |  |  |  |  |  |
| Below threshold |  | 1.96** |  | 1.78** |  | 2.83** |
|  |  | (1.69, 2.27) |  | (1.55, 2.01) |  | (2.37, 3.39) |
| 1 to 1.99 x’s above |  | 1.87** |  | 1.67** |  | 2.68** |
|  |  | (1.65, 2.11) |  | (1.49, 1.88) |  | (2.30, 3.13) |
| 2 to 2.99 x’s above |  | 1.79** |  | 1.66** |  | 2.24** |
|  |  | (1.60, 1.99) |  | (1.50, 1.83) |  | (1.94, 2.58) |
| 3 to 3.99 x’s above |  | 1.34** |  | 1.25** |  | 1.74** |
|  |  | (1.20, 1.49) |  | (1.14, 1.48) |  | (1.50, 2.01) |
| 4 to 4.99 x’s above |  | 1.31** |  | 1.30** |  | 1.50** |
|  |  | (1.18, 1.46) |  | (1.18, 1.43) |  | (1.29, 1.75) |
| Employment Status (not working) |  |  |  |  |  |  |
| Working Less Than Full Time |  | 0.67** |  | 0.72** |  | 0.47** |
|  |  | (0.61, 0.75) |  | (0.65, 0.80) |  | (0.41, 0.54) |
| Working Full Time |  | 0.52** |  | 0.61** |  | 0.37** |
|  |  | (0.48, 0.57) |  | (0.56, 0.66) |  | (0.33, 0.41) |
| Health Coverage (none) |  |  |  |  |  |  |
| Public Health Coverage |  | 1.55** |  | 1.51** |  | 1.81** |
|  |  | (1.36, 1.76) |  | (1.33, 1.71) |  | (1.55, 2.11) |
| Private Health Coverage |  | 0.96 |  | 0.97 |  | 0.73** |
|  |  | (0.86, 1.08) |  | (0.87, 1.077) |  | (0.63, 0.85) |
| Other Coverage |  | 1.08 |  | 1.04 |  | 0.81ϯ |
|  |  | (0.89, 1.31) |  | (0.86, 1.26) |  | (0.64, 1.01) |
| Has a Usual Place for Care (no) |  | 1.01 |  | 1.06 |  | 1.10 |
|  |  | (0.90, 1.13) |  | (0.95, 1.17) |  | (0.93, 1.30) |
| Has Seen a Provider in Past Year (no) |  | 1.44** |  | 1.43** |  | 2.04** |
|  |  | (1.32, 1.57) |  | (1.33, 1.55) |  | (1.76, 2.35) |
| BMI Categories (normal) |  |  |  |  |  |  |
| Underweight |  | 1.08 |  | 1.03 |  | 2.30** |
|  |  | (0.76, 1.53) |  | (0.74, 1.44) |  | (1.51, 3.49) |
| Overweight |  | 1.09* |  | 1.08ϯ |  | 1.13* |
|  |  | (1.01, 1.19) |  | (0.99, 1.16) |  | (1.01, 1.27) |
| Obese |  | 1.50** |  | 1.36** |  | 2.04** |
|  |  | (1.38, 1.64) |  | (1.26, 1.47) |  | (1.83, 2.28) |
| BMI Info Missing |  | 1.40** |  | 1.31** |  | 3.04** |
|  |  | (1.24, 1.57) |  | (1.17, 1.47) |  | (2.61, 3.53) |
| Smoking Status (never) |  |  |  |  |  |  |
| Former |  | 1.42** |  | 1.46** |  | 1.44** |
|  |  | (1.31, 1.53) |  | (1.36, 1.56) |  | (1.31, 1.59) |
| Current |  | 1.89** |  | 1.86** |  | 2.03** |
|  |  | (1.72, 2.01) |  | (1.71, 2.02) |  | (1.82, 2.26) |
| Female |  | 1.55** |  | 1.61** |  | 0.85** |
|  |  | (1.45, 1.65) |  | (1.52, 1.71) |  | (0.78, 0.93) |
| Age |  | 1.04** |  | 1.04* |  | 1.18** |
|  |  | (1.01, 1.07) |  | (1.02, 1.07) |  | (1.13, 1.22) |
| Age-Squared |  | 0.99** |  | 0.99** |  | 0.99** |
|  |  | (0.99, 1.00) |  | (0.99, 1.00) |  | (0.98, 0.99) |
| Survey year (2019) |  |  |  |  |  |  |
| 2020 |  | 1.03 |  | 1.09ϯ |  | 0.90 |
|  |  | (0.94, 1.13) |  | (1.00, 1.19) |  | (0.79, 1.03) |
| 2021 |  | 1.14** |  | 1.16** |  | 0.88* |
|  |  | (1.05, 1.25) |  | (1.07, 1.25) |  | (0.79, 0.98) |
| 2022 |  | 1.20** |  | 1.23** |  | 0.94 |
|  |  | (1.10, 1.31) |  | (1.14, 1.33) |  | (0.84, 1.05) |
| US Citizen |  | 1.11 |  | 1.11 |  | 1.05 |
|  |  | (0.81, 1.53) |  | (0.82, 1.50) |  | (0.68, 1.64) |
| Marital Status (currently married) |  |  |  |  |  |  |
| Divorced/Separated/Widowed |  | 1.52** |  | 1.39** |  | 1.14* |
|  |  | (1.40, 1.65) |  | (1.28, 1.50) |  | (1.03, 1.27) |
| Never Married |  | 1.38** |  | 1.30** |  | 1.10 |
|  |  | (1.26, 1.51) |  | (1.20, 1.41) |  | (0.97, 1.26) |
| Minor at Home |  | 0.64** |  | 0.78** |  | 0.62** |
|  |  | (0.59, 0.71) |  | (0.73, 0.85) |  | (0.56, 0.70) |
| Length at Current House |  | 0.93** |  | 0.93** |  | 0.96* |
|  |  | (0.90, 0.96) |  | (0.90, 0.95) |  | (0.92, 0.99) |
| US region (northeast) |  |  |  |  |  |  |
| North Central/Midwest |  | 1.06 |  | 0.96 |  | 1.10 |
|  |  | (0.95, 1.18) |  | (0.88, 1.05) |  | (0.96, 1.27) |
| South |  | 0.97 |  | 0.95 |  | 1.23** |
|  |  | (0.88, 1.08) |  | (0.87, 1.04) |  | (1.08, 1.40) |
| West |  | 1.26** |  | 1.15** |  | 1.22** |
|  |  | (1.11, 1.42) |  | (1.04, 1.27) |  | (1.06, 1.40) |
| N | 41,752 | 41,752 | 41,752 | 41,752 | 41,752 | 41,752 |
| AIC | 41,125 | 37,103 | 46,627 | 42,957 | 29,091 | 22,243 |
| BIC | 41,154 | 37,398 | 46,657 | 43,253 | 29,120 | 22,543 |

*Note*: Odds ratios with 95% confidence intervals (CI). All estimates are weighted. Reference categories included in (parentheses).

** p < 0.01, * p < 0.05, ϯ p < 0.10

Table S3: Multivariable results predicting diagnosed health conditions among Whites by nativity status, ages 25-64: National Health Interview Survey, United States, 2019-2022

|  | Diabetes  OR (95% CI) | | Hypertension  OR (95% CI) | | COPD  OR (95% CI) | |
| --- | --- | --- | --- | --- | --- | --- |
|  | Model 1 | Model 2 | Model 1 | Model 1 | Model 2 | Model 1 |
| (Intercept) | 0.07** | 0.00** | 0.36** | 0.00** | 0.04** | 0.00** |
|  | (0.06, 0.07) | (0.00, 0.00) | (0.35, 0.37) | (0.00, 0.00) | (0.04, 0.05) | (0.00, 0.00) |
| Nativity (US-born) |  |  |  |  |  |  |
| Foreign-Born | 0.79* | 1.07 | 0.70** | 0.78** | 0.57** | 0.88 |
|  | (0.63-0.99) | (0.81-1.41) | (0.62-0.79) | (0.66-0.91) | (0.42-0.79) | (0.60-1.29) |
| Educational Attainment |  |  |  |  |  |  |
| HS Diploma |  | 0.78* |  | 0.86ϯ |  | 0.61** |
|  |  | (0.63, 0.96) |  | (0.73, 1.02) |  | (0.49, 0.76) |
| Some College or Associate |  | 0.82ϯ |  | 0.80** |  | 0.66** |
|  |  | (0.66, 1.02) |  | (0.68, 0.94) |  | (0.52, 0.82) |
| College Degree or More |  | 0.58** |  | 0.70** |  | 0.47** |
|  |  | (0.46, 0.72) |  | (0.59, 0.83) |  | (0.37, 0.61) |
| Poverty (5 x’s or more above threshold) |  |  |  |  |  |  |
| Below threshold |  | 1.57** |  | 1.52** |  | 2.15** |
|  |  | (1.23, 2.01) |  | (1.30, 1.77) |  | (1.64, 2.81) |
| 1 to 1.99 x’s above |  | 1.52** |  | 1.25** |  | 2.13** |
|  |  | (1.27, 1.82) |  | (1.11, 1.41) |  | (1.67, 2.70) |
| 2 to 2.99 |  | 1.50** |  | 1.28** |  | 1.66** |
|  |  | (1.26, 1.79) |  | (1.16, 1.40) |  | (1.30, 2.14) |
| 3 to 3.99 |  | 1.40** |  | 1.14** |  | 1.62** |
|  |  | (1.19, 1.64) |  | (1.03, 1.25) |  | (1.30, 2.03) |
| 4 to 4.99 |  | 1.26** |  | 1.15** |  | 1.60** |
|  |  | (1.08, 1.47) |  | (1.05, 1.26) |  | (1.26, 2.02) |
| Employment Status (not working) |  |  |  |  |  |  |
| Working Less Than Full Time |  | 0.74** |  | 0.81** |  | 0.67** |
|  |  | (0.61, 0.89) |  | (0.72, 0.90) |  | (0.54, 0.83) |
| Working Full Time |  | 0.82** |  | 0.84** |  | 0.70** |
|  |  | (0.72, 0.93) |  | (0.77, 0.91) |  | (0.60, 0.82) |
| Health Coverage (none) |  |  |  |  |  |  |
| Public Health Coverage |  | 1.83** |  | 1.18* |  | 2.08** |
|  |  | (1.37, 2.45) |  | (1.02, 1.38) |  | (1.63, 2.65) |
| Private Health Coverage |  | 1.03 |  | 0.94 |  | 0.88 |
|  |  | (0.79, 1.35) |  | (0.83, 1.08) |  | (0.68, 1.15) |
| Other Coverage |  | 0.80 |  | 0.91 |  | 1.22 |
|  |  | (0.54, 1.18) |  | (0.74, 1.11) |  | (0.87, 1.72) |
| Has a usual place for care (no) |  | 1.65** |  | 1.60** |  | 1.22 |
|  |  | (1.14, 2.40) |  | (1.40, 1.83) |  | (0.92, 1.62) |
| Has seen a provider in past year (no) |  | 4.32** |  | 2.76** |  | 1.81** |
|  |  | (3.30, 5.66) |  | (2.48, 3.07) |  | (1.43, 2.29) |
| BMI Categories (normal) |  |  |  |  |  |  |
| Underweight |  | 0.75 |  | 0.92 |  | 1.27 |
|  |  | (0.25, 2.23) |  | (0.57, 1.49) |  | (0.65, 2.46) |
| Overweight |  | 1.71** |  | 1.90** |  | 1.02 |
|  |  | (1.42, 2.05) |  | (1.75, 2.08) |  | (0.85, 1.21) |
| Obese |  | 4.25** |  | 3.76** |  | 1.48** |
|  |  | (3.59, 5.04) |  | (3.47, 4.07) |  | (1.26, 1.74) |
| BMI Info Missing |  | 4.78** |  | 3.57** |  | 1.88** |
|  |  | (3.90, 5.86) |  | (3.20, 3.99) |  | (1.51, 2.34) |
| Smoking Status (never) |  |  |  |  |  |  |
| Former |  | 1.04 |  | 1.86** |  | 2.97** |
|  |  | (0.93, 1.17) |  | (1.11, 1.27) |  | (2.50, 3.53) |
| Current |  | 0.91 |  | 1.28** |  | 5.18** |
|  |  | (0.78, 1.06) |  | (1.18, 1.39) |  | (4.38, 6.12) |
| Female |  | 0.68** |  | 0.63** |  | 1.29** |
|  |  | (0.61, 0.75) |  | (0.59, 0.67) |  | (1.13, 1.48) |
| Age |  | 1.22** |  | 1.11** |  | 1.14** |
|  |  | (1.15, 1.30) |  | (1.08, 1.14) |  | (1.07, 1.21) |
| Age-Squared |  | 0.99** |  | 0.99** |  | 0.99* |
|  |  | (0.98, 0.99) |  | (0.99, 1.00) |  | (0.99, 1.00) |
| Year (2019) |  |  |  |  |  |  |
| 2020 |  | 0.84* |  | 0.96 |  | 1.17ϯ |
|  |  | (0.73, 0.98) |  | (0.87, 1.04) |  | (0.98, 1.41) |
| 2021 |  | 1.14ϯ |  | 1.04 |  | 1.05 |
|  |  | (1.00, 1.30) |  | (0.96, 1.12) |  | (0.90, 1.23) |
| 2022 |  | 1.06 |  | 1.01 |  | 1.04 |
|  |  | (0.93, 1.21) |  | (0.94, 1.10) |  | (0.88, 1.23) |
| US Citizen |  | 1.20 |  | 0.97 |  | 1.67 |
|  |  | (0.66, 2.18) |  | (0.70, 1.34) |  | (0.69, 4.07) |
| Marital Status (currently married) |  |  |  |  |  |  |
| Divorced/Separated/Widowed |  | 0.94 |  | 1.07ϯ |  | 1.13 |
|  |  | (0.82, 1.08) |  | (0.99, 1.16) |  | (0.96, 1.33) |
| Never Married |  | 1.12 |  | 0.96 |  | 0.94 |
|  |  | (0.97, 1.31) |  | (0.88, 1.05) |  | (0.77, 1.15) |
| Child <18yrs present in home |  | 0.75** |  | 0.83** |  | 0.76* |
|  |  | (0.65, 0.88) |  | (0.77, 0.89) |  | (0.62, 0.92) |
| Length at current house/apt |  | 0.95** |  | 1.00 |  | 0.93** |
|  |  | (0.91, 0.99) |  | (0.97, 1.02) |  | (0.88, 0.98) |
| US region (northeast) |  |  |  |  |  |  |
| North Central/Midwest |  | 1.30** |  | 1.05 |  | 1.33** |
|  |  | (1.12, 1.51) |  | (0.96, 1.14) |  | (1.08, 1.64) |
| South |  | 1.30** |  | 1.23** |  | 1.39** |
|  |  | (1.12, 1.51) |  | (1.13, 1.35) |  | (1.13, 1.72) |
| West |  | 0.94 |  | 1.03 |  | 1.20 |
|  |  | (0.79, 1.12) |  | (0.93, 1.14) |  | (0.95, 1.52) |
| N | 41,752 | 41,752 | 41,752 | 41,752 | 41,752 | 41,752 |
| AIC | 19,568 | 16,222 | 47,905 | 40,100 | 14,305 | 11,045 |
| BIC | 19,598 | 16,518 | 47,934 | 40,398 | 14,335 | 11,348 |

*Note*: Odds ratios with 95% confidence intervals (CI). All estimates are weighted. Reference categories included in (parentheses).

** p < 0.01, * p < 0.05, ϯ p < 0.10
